# ZnO-dotted porous ZnS cluster microspheres for high efficient, Pt-free photocatalytic hydrogen evolution

**DOI:** 10.1038/srep08858

**Published:** 2015-03-09

**Authors:** Aiping Wu, Liqiang Jing, Jianqiang Wang, Yang Qu, Ying Xie, Baojiang Jiang, Chungui Tian, Honggang Fu

**Affiliations:** 1 Key Laboratory of Functional Inorganic Material Chemistry, Ministry of Education of the People's Republic of China, Heilongjiang University, Harbin 150080 (P. R. China); 2 Shanghai Synchrotron Radiation Facility (SSRF), Shanghai Institute of Applied Physics, Chinese Academy of Sciences, Shanghai 201204, China

## Abstract

The Pt-free photocatalytic hydrogen evolution (PHE) has been the focus in the photocatalysis field. Here, the ZnO-dotted porous ZnS cluster microsphere (PCMS) is designed for high efficient, Pt-free PHE. The PCMS is designed through an easy “controlling competitive reaction” strategy by selecting the thiourea as S^2−^ source and Zn(Ac)_2_**·**2H_2_O as Zn source in ethylene glycol medium. Under suitable conditions, one of the PCMS, named PCMS-1, with high S_BET_ specific area of 194 m^2^g^−1^, microsphere size of 100 nm and grain size of 3 nm can be obtained. The formation of PCMS is verified by TEM, XAES, XPS, Raman and IR methods. Importantly, a series of the experiments and theoretical calculation demonstrate that the dotting of ZnO not only makes the photo-generated electrons/hole separate efficiently, but also results in the formation of the active catalytic sites for PHE. As a result, the PCMS-1 shows the promising activity up to 367 μmol h^−1^ under Pt-free condition. The PHE activity has no obvious change after addition 1 wt.% Pt, implying the presence of active catalytic sites for hydrogen evolution in the PCMS-1. The easy synthesis process, low preparation cost of the PCMS makes their large potential for Pt-free PHE.

Photocatalytic hydrogen evolution (PHE) has received the intensive attention because it provides an attractive solution to the globe energy and environmental problems^1^. To effectively produce H_2_, the band gap energy (Eg) of a semiconductor should be > 1.23 eV with more negative bottom of the conduction band (CB) than the reduction potential of H+ to H_2_^2^. Many catalysts match the demand, but usually show poor activity, mainly due to the easy recombination of the photogenerated charges (e^−^)/holes (h^+^) and the lack of the active catalytic sites^3^. The noble metal (Pt, Au, Pd etc.) can trap easily the electrons due to its low Fermi level. When compounding with semiconductors, they can serve as electron sinks via the formation of a Schottlky junction and provide effective proton reductions sites for hydrogen evolution with a low kinetic activation barrier^4^,^5^. However, the noble metal is scarce and expensive, which largely hinders their practical application.

Several strategies have been proposed to enhance the PHE activity, such as changing the morphology of the photocatalyst^6^,^7^,^8^, the reduction with H_2_ to form mixed valence^9^,^10^ and compounding with carbon^11^. The heterojunction from integrating two or more semiconductors with matched energy band can optimize the capture of light, promote the separation of photogenerated e^−^/h^+^ through creating a built-in electric field in the space-charge region, and hence enhance the PHE activity obviously^12^,^13^. Typically, the photocatalytic activity of TiO_2_ can be enhanced up to four times through the deposition of anatase nanoparticles (NPs) on the surface of rutile to form an anatase/rutile TiO_2_ surface-phase heterjunction^14^. The MgTiO_3_–MgTi_2_O_5_ heterogeneous nanorods can give the enhanced PHE activity significantly under UV-light^15^. However, in these systems, the Pt cocatalyst needs to be used to provide the active catalytic sites, and thus to promote the PHE activity. The development of the highly efficient and low-cost noble-metal free cocatalysts that can both promote the separation of photogenerated e^−^/h^+^ and provide active sites are needed to realize real Pt-free PHE. Until now, the non-Pt cocatalysts have mainly concentrated in the layer disulphide, such as MoS_2_ and WS_2_^16^,^17^,^18^,^19^,^20^,^21^. For example, the MoS_2_/graphitic carbon nitride (g-GN) can give the comparable PHE activity with corresponding Pt/g-GN^19^. The sequential grown of TiO_2_ and MoS_2_ on graphene can give a ternary TiO_2_-MoS_2_/graphene catalyst that shows a H_2_ production rate of 165.3 μmol h^−1^ under UV-light irradiation^17^. The results have indicated the large possibility to replace noble Pt with cheap materials through designing the proper structure. However, there is less success in searching other alternative to Pt, besides the layer disulphide. In addition, the origin of active like-Pt catalytic sites has needed to be deeply studied for the Pt-free PHE. It is important to construct a new, non-disulphide system for Pt-free PHE and to elucidate the origin of active like-Pt catalytic sites.

ZnS is one of the important II-VI group semiconductors with promising application in the photocatalytic field. The energy gap of ZnS is 3.7 eV with a CB of −1.04 eV^22^, more negative than reduction potential of H^+^ to H_2_. So, ZnS is a suitable catalyst for PHE in principle. The studies have indicated the certain activity of ZnS for PHE, but the activity needs to be further enhanced by the assistance of Pt and Au^23^,^24^. It is proposed to integrate ZnS with other materials to form a heterostructure for accelerating the separation of the photogenerated e^−^/h^+^ based on the virtues of the heterostructure. Zinc oxide (ZnO), another semiconductor containing Zn element, has a relatively positive position of conduction band (−0.31 eV) and valence band (2.89 eV) compared with ZnS. Thus, the combination of ZnO with ZnS is helpful to accelerate the separation of e^−^/h^+^ for improving the PHE activity due to their matched energy band (Figure 1a). Recent two works have demonstrated that the combination of ZnO and ZnS can catalyze hydrogen evolution even without Pt co-catalysts^25^,^26^. However, the large size of both ZnS and ZnO in the composites can result in less contact of the catalyst with agents, thus not favorable for promoting the catalytic activity of the catalyst^27^. Especially, the origin of active catalytic sites for hydrogen evolution is not elucidated. Based on the principle of the heterogeneous catalysis, the activity will be largely enhanced by the high dispersion of ZnO on ZnS, typically, by the formation of ZnO-dotted ZnS. Therefore, it is designed and necessary to build a ZnO/ZnS heterjunction for highly effective, Pt-free PHE, and to elucidate the origin of active catalytic sites for hydrogen production.

In this paper, we have reported the synthesis of ZnO-dotted porous ZnS cluster microsphere (PCMS) with porous structure, high specific area and small grain size through an easy “controlling competitive reaction” strategy in ethylene glycol (EG). The thiourea is used as S^2−^ source and zinc acetate is used as Zn source. The S^2−^ from thiourea can react with Zn^2+^ to form ZnS. Also, the ZnO can form in EG via “forced hydrolysis” process^28^. The competition between the formation of ZnO and ZnS can result in the formation of ZnO-dotted ZnS through one-pot process by simply tuning the ratio of thiourea and zinc acetate. The synthesis of PCMS is easy and simple, while the syntheses of most heterjunctions in previous work are slightly sophisticated in order to achieve desired structures. The formation of PCMS is verified by the combination of XRD, TEM, XAES, XPS, Raman and IR methods. Importantly, the origin of Pt-free PHE is also elucidated by combining a series of the experiments and theoretical calculation. That is, the ZnO dotting not only makes the photo-generated e^−^/h^+^ separate efficiently, but also results in the formation of the active catalytic sites for PHE just like the Pt modification. The PCMS-1 shows the promising activity up to 367 μmol h^−1^ under Pt-free condition. The easy synthesis process, low preparation cost of the PCMS makes the large potential for Pt-free PHE.

## Results and Discussion

### The Structure Characterization of PCMS

The PCMS were prepared through an easy one-pot “controlling competitive reaction” strategy in EG. A typical sample, PCMS-1, was prepared at 180°C for 12 h with MR 1:1 of thiourea and Zn(Ac)_2_. The XRD pattern of PCMS-1 can be indexed to the hexagonal zinc sulfide (No. 80-0007), and no other phase is found (Figure 2a). The size of the sphere is about 100 nm with good uniform both in the size and morphology (Figure 2b). The images in Figure 2c, d confirm that the spheres are composed of the small clusters of 3 nm. The small grain is favorable for the transfer of e^−^ from the interior to surface of catalysts, thus enhancing the PHE activity^29^. The high-resolution TEM image (Figure 2d) shows the lattice fringe of 0.31 nm that is an inter-planar distance of the (002) plane of hexagonal ZnS. In addition, the pores from the accumulation of the small particle can be observed. The porous structure and small size of particles implies the high surface area of the PCMS-1. Indeed, the N_2_ adsorption-desorption test indicates a high BET surface area (S_BET_) of 194 m^2^/g of the sample (Figure S1 and Table S1). The porous structure with high S_BET_ can provide the highly accessible interface for efficient injection of photo-generated e^−^, and allow light-scattering inside the pore, thus enhance the activity of materials^30^,^31^. In present system, the presence of ZnO is quite possible because of the formation of ZnO and ZnS is competive reaction. However, no evidence for the formation of the ZnO is observed both in XRD and TEM tests. This should be due to the very small size and/or amount of ZnO components that are difficult to be detected by TEM and XRD test. Therefore, the X-ray energy dispersive spectrometry (EDS) element mapping is done to analyze the element composition of the PCMS-1. The EDS mapping indicates the homogeneous distribution of Zn, S and O elements throughout the sphere, implying the presence of ZnO in the PCMS-1 homogeneously (Figure S2). By changing the MR value, we can also obtain the PCMS with similar microstructure and crystalline orientation. Typically, the porous microspheres with sphere size of 70 nm and grain size of 5 nm can be obviously seen in PCMS-2.5 sample (Figure 2f to i).

To further demonstrate the presence of ZnO (Zn-O) in the PCMS-1, consequentially, the formation of ZnO-dotted structure, the XPS, Raman, IR and XAES test are performed because their high sensitivity to analyze the Zn-O or O. As shown in Figure 3a, the peaks belonging to O, S and Zn can be clearly seen in the wide scan spectrum, implying the presence of ZnO and ZnS in PCMS-1. Especially, the O1s XPS spectra can be deconvoluted into the three peaks (Figure 3b), in which the peak at 530.4 eV can be ascribed to the oxygen (OL) of ZnO^32^, which is a indicative of the presence of ZnO. The high resolution XPS spectrum in Figure S3 indicates that Zn 2p1/2 and 2p3/2 are located at 1042.95 eV and 1019.9 eV, which are characteristics for Zn^2+^ 33. In IR spectrum (Figure S4), a typical vibration of Zn–O bond in ZnO^34^ can be seen at about 500 cm^−1^ with noticeable intensity, indicating the presence of ZnO in the PCMS-1. Raman is a powerful tool for analyzing the structure of oxides. From the Figure S5, two obvious peaks can be observed at 557 cm^−1^ and 1126 cm^−1^. The peak at 557 cm^−1^ can be assigned to the E1 (LO) vibration model of hexagonal wurtzite structure ZnO crystal. While the peak at 1126 cm^−1^ is due to multiple phonon scattering processes of Zn-O. The peaks located at 420 cm^−1^ is from the E2 (high) vibration model of Zn-O^35^. The normalized XAES of PCMS-1 and corresponding EXAFS (extended x-ray absorption fine structure) are provided to give the further insight. The corresponding Fit parameters are shown in Table 1. We can see that the N value of Zn-S (N = 3.5) in PCMS-1 is close to that of standard ZnS (S-ZnS) (N = 4), indicating the existence of ZnS as the major phase of PCMS-1. The slight low N value of Zn-S (3.5 vs 4) also implies the presence of slight ZnO in PCMS-1. In addition, the lower N value of Zn-S-Zn (4.5) for PCMS-1 than that for S-ZnS (12) implies the small size of ZnS grain in PCMS-1. Notably, the Zn-O can be observed in PCMS-1 with N value of 1, more lower than 3.9 in bulk ZnO^36^. Generally, the low N value implies the small size of the particles. Typically, for single atom Ag chain, the N is 2, more lower than 12 for bulk Ag^37^. A single atom Pt has no shown any Pt–Pt contribution^38^. Therefore, the low N value of Zn-O implies the partial replacement of surface S atom in ZnS by O, and the formation of the ZnO-dotted ZnS clusters.

The studies have indicated that the nitrogen-containing organic compounds can potentially transfer to carbon nitride based materials under solvothermal reaction condition. For example, Cui and co-worker demonstrated that the conjugated carbon nitride could be obtained by polymerization of cyanuric chloride and melamine in acetonitrile solvent^39^. The characteristic peaks of triazine units in carbon nitride based materials are located at about 810 cm^−1^ 40,41. However, IR spectrum of PCMS-1 sample have not shown the characteristic peak of triazine units in carbon nitride. In addition, in XRD patterns, we cannot observe any peaks belonging to the carbon nitride (the peaks located at about 12.5° and 27.5°) in the samples. The test indicated that no carbon nitride based materials existed in PCMS samples.

The combination of TEM, XRD, Raman, IR and XAES indicates the formation of ZnO-dotting porous ZnS cluster microsphere. The homogeneous distribution of Zn, S and O elements throughout the sphere can also be observed from EDS mapping test. Therefore, it can be concluded that dotting of ZnO on ZnS is uniform. The formation of PCMS can be contributed into the large difference (more than 10^6^) in the equilibrium concentration (EC) of Zn^2+^ for the ZnS and ZnO (Zn(OH)_2_). There are several main reactions in present reaction system:
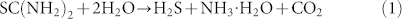

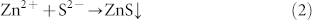

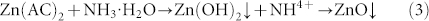


The ZnO can also be formed via following reactions:
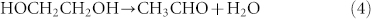

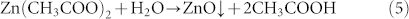


**Figure 1:**
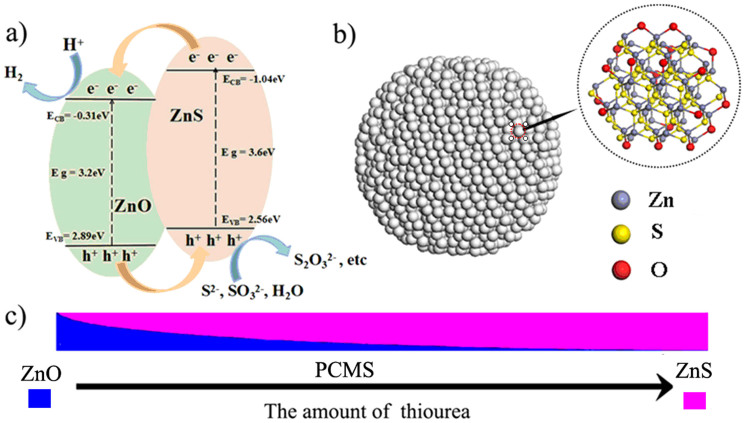
(a) The energy band structure of ZnO/ZnS heter-junctions; (b) the schematic structure of ZnO-dotted ZnS; (c) the structure evolution from ZnO to PCMS.

**Figure 2:**
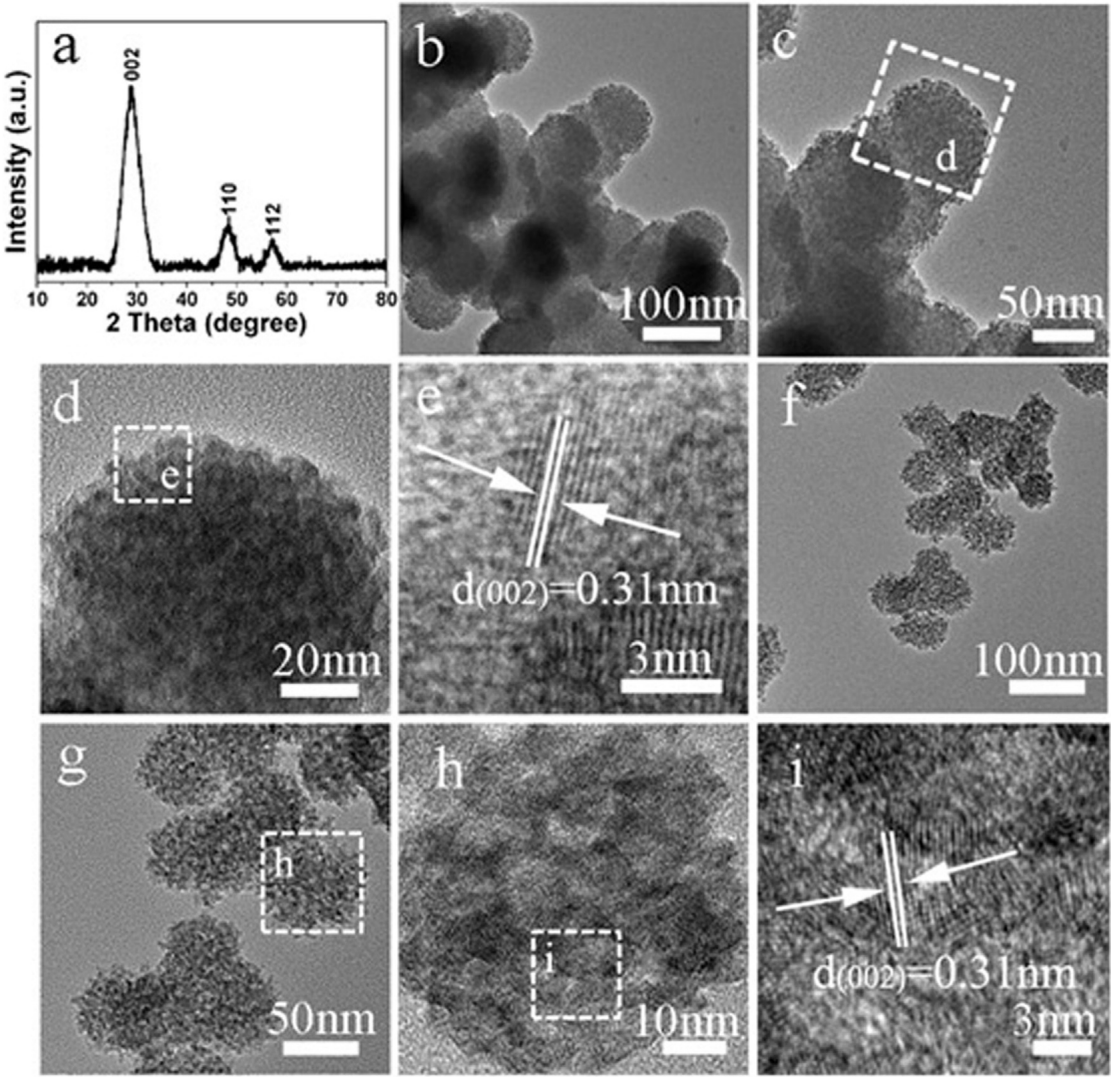
(a) XRD pattern, (b–e) TEM and HRTEM of PCMS-1; the f-i are TEM and HRTEM images of PCMS-2.5.

**Figure 3:**
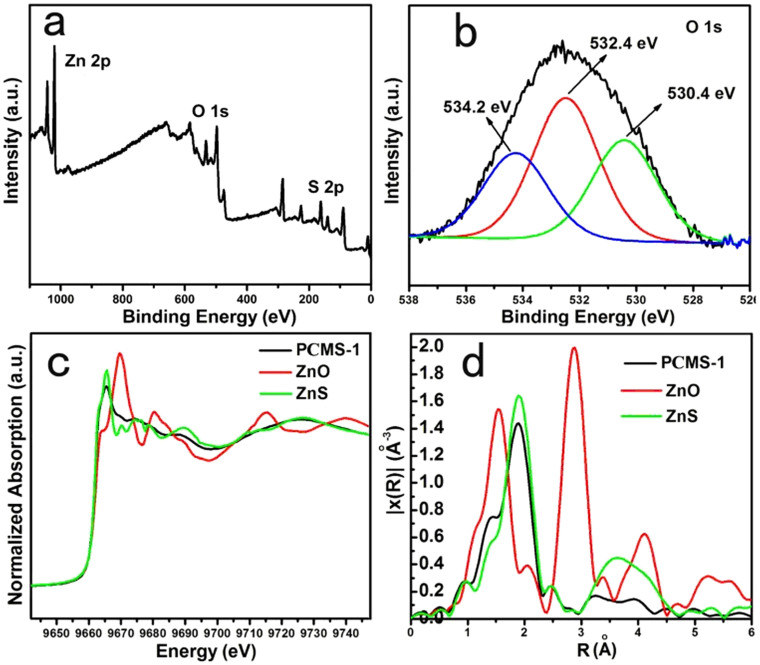
(a) the wide scan XPS spectrum and (b) the O1s XPS spectrum of PCMS-1, (c) XAES and (d) corresponding EXAFS spectra of PCMS-1.

In this system, the Zn^2+^ can react with S^2−^ and OH^−^ (NH_3_) from Reaction 1 (R1) to form ZnS (R2) and ZnO (R3). So, the formation of ZnS and ZnO are competitive reaction. The Ksp of ZnS is about 1.6 × 10^−24^ (the EC of Zn^2+^ is 1.26 × 10^−12^ mol L^−1^), while that for Zn(OH)_2_, a intermedium for the formation of ZnO, is about 1.2 × 10^−17^ (the EC of Zn^2+^ is 1.46 × 10^−6^ mol L^−1^). So, ZnS particles will form preferentially as its very low Ksp and low EC. Followed this, the OH^−^ provided by NH_3_ (R1) and R4 (the reaction can result in the slow release of H_2_O) will react with preformed ZnS to form ZnO (R3 and R5). Although OH^−^ source provided by R1 and R4 is largely excessive over S^2−^, the large difference in EC of Zn^2+^ for the ZnS and ZnO makes that only very small amount of S^2−^ in ZnS can be replaced by O in-situ to form ZnO-dotted ZnS. The amount of ZnO is very little, especially, in the case of high S/Zn ratio due to the large difference in the EC of Zn^2+^ for the ZnS and ZnO, thus leading to the formation of ZnO-dotted ZnS microspheres. Therefore, the ZnO cannot be found by TEM and XRD, and can only be detected by more sensitive spectra techniques, such as XPS, IR, Raman and XAES. Based on the R 1-5, the amount of ZnO and ZnS in the products can be tuned by changing the MR values (Figure 1c). A series of the experiments also demonstrate the evolution from ZnO to ZnO-dotted ZnS. In detail, the ZnO can be formed as no thiourea was used in the synthesis as shown by XRD in Figure S6a. For the PCMS-0.5 prepared at MR = 1/2, both ZnO and ZnS phase can be observed as low amount of S source. The presence of ZnO can be verified by XRD and Raman (Figure S6b and S7a). Meantime, it can be found that, in this case, the peaks ascribed to ZnS are more intensive than those to ZnO, indicating that the ZnS are main phase of PCMS-0.5. In the case of MR = 1 (PCMS-1), the ZnO-dotted porous ZnS cluster microspheres can be formed. Although the PCMS-2.5 show similar XRD pattern (Figure S6c) with PCMS-1, only very weak peaks corresponding to ZnO can be observed in Raman spectra (Figure S7b), indicating the more low amount of ZnO in the sample. The PCMS-5 sample shows similar XRD patterns with PCMS-1 (Figure S6d) but no obvious Raman variation of ZnO can be found (Figure S7c). In addition, in IR spectra of PCMS-2.5 and PCMS-5, the peak corresponding to Zn-O vibration is not obvious (Figure S8). The results indicate the decrease of ZnO amount with the increase of MR value. We can also observe the obvious effect of MR value on the micro- structure of the samples. The size of the microspheres decreases with the increase of MR values. The size is concentrated in 160 nm, 100 nm, 70 nm and 40 nm for PCMS-0.5, PCMS-1, PCMS-2.5 and PCMS-5, respectively (Figure 2 and Figure S9). In contrast, the size of small grain that composed of PCMS give an opposite change. The grain size is about 2 nm, 3 nm, 5 nm and 8 nm for PCMS-0.5, PCMS-1, PCMS-2.5 and PCMS-5 (Figure 2 and Figure S9). The change of grain size and ZnO amount can also be reflected by the XAES and EXAFS (Table 1). For PCMS-0.5, the N value of Zn-O is about 1.7, ascribing to the relative high amount of ZnO. The N value of Zn-S for PCMS-0.5 is 2.3, more lower than Standard ZnS. The result indicates that the co-existence of ZnS and ZnO and the content of ZnO in PCMS-0.5 is higher than in PCMS-1. The N values of Zn-S for PCMS-2.5 and PCMS-5 are same with that for Standard ZnS. The results indicate the existence of ZnS as major phase of PCMS-2.5 and PCMS-5. However, the EXAFS data do not reveal obvious Zn–O contribution for PCMS-5 and PCMS-2.5, indicating very low amount of ZnO in the samples. The N values of Zn-S-Zn are in the order of PCMS-1 (4.5) < PCMS-2.5 (5) < PCMS-5 (6), implying the increase of grain size in the order of PCMS-1 < PCMS-2.5< PCMS-5. The result is consistent with that of XRD and TEM results. The tunable microstructure provides a chance for tuning the performance of PCMS. The combination of above analysis with EXAFS and EDS indicated that the formation of ZnO-dotted ZnS cluster microspheres, and the ZnO should be supported on the surface of ZnS, but not mixed randomly with ZnS component.

### The Test of PHE Activity of PCMS

**Figure 4:**
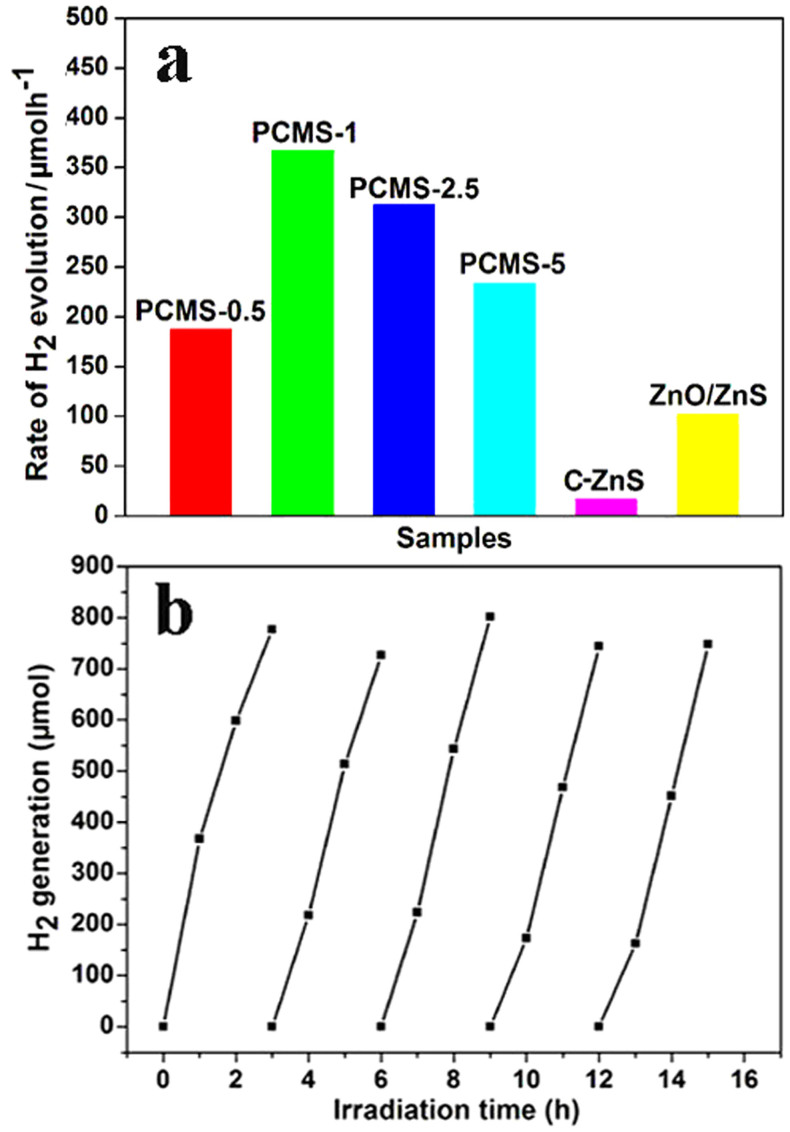
(a) The PHE activity of PCMS, C-ZnS and ZnO/ZnS; (b) the recycled PHE activity of PCMS-1. The ZnO/ZnS is prepared according to the Ref. 25.

The promising characteristics of PCMS, including the uniform dotting of ZnO on ZnS, the porous structure and small crystallite size are favorable for its application in PHE. The PHE activity of PCMS was evaluated under UV-light irradiation without using Pt cocatalyst, and the results are shown in Figure 4a. We can see that all PCMS samples show obvious activity and the PCMS-1 sample gives the highest one. The H_2_-production rate is about 367 μmol h^−1^, 188 μmol h^−1^, 312 μmol h^−1^ and 234 μmol h^−1^ for PCMS-1, PCMS-0.5, PCMS-2.5 and PCMS-5, respectively (Figure 4a). The activity of PCMS samples is higher than that of some Pt-free photocatalysts, for example, ternary TiO_2_-MoS_2_/graphene (165.3 μmol h^−1^)^17^. However, the commercial ZnS (C-ZnS) only show neglectable PHE activity (17 μmol h^−1^) in identical conditions. As a control, we have also synthesized the rod-like ZnO/ZnS rods according to the Ref. 25. The PHE activity of the ZnO/ZnS rods is about 110 μmol h^−1^. The activity of PCMS-1 is about 3 times higher than that of the ZnO/ZnS rods. The lower activity of ZnO/ZnS rods should be relative with the larger size of ZnO and ZnS in the composites. The results indicate that the formation of the ZnO-dotted porous ZnS structure is favorable for promoting the PHE activity largely.

We know that the activity of the materials is governed by their microstructure, such as S_BET_, grain size and relative content of different composites. Generally, the high S_BET_ can provide the more contact of agents with the photocatalyst, thus is favorable for the improvement of the activity of the materials in principle. In addition, more small grain size, more easy transfer of e^−^ from interior to surface of catalysts, thus enhances the PHE activity of catalyst. In the PCMS, the ZnS is light-adsorption components and dotting of ZnO can result in the formation of the active catalytic sites. So, the content of ZnS and dotted ZnO is important factor that affect the activity of the PCMS. The S_BET_ values are about 105.5, 194.6, 265.5 and 135.9 m^2^/g for PCMS-0.5, PCMS-1, PCMS-2.5 and PCMS-5, respectively. From the viewpoint of S_BET_, the PCMS-2.5 should have highest activity in principle, but it activity is lower than PCMS-1 with lower S_BET_ value in test. So, the factors of grain size and ZnO content must be considered. Based on TEM and XAES results, the grain sizes are in the order of PCMS-0.5 (2 nm) < PCMS-1(3 nm) < PCMS-2.5 (5 nm) < PCMS-5 (8 nm). At the same time, the content of ZnO decreases in the order of PCMS-0.5 > PCMS-1 > PCMS-2.5 > PCMS-5. It can be seen that the PCMS-1 sample gives the higher activity than PCMS-0.5 with higher amount of ZnO, PCMS-2.5 and PCMS-5 having lower amount of ZnO. The low activity of PCMS-0.5 should be relative with its lower BET surface (105 m^2^/g) than PCMS-1 (194 m^2^/g). In addition, we think that the high content of ZnO is not favorable for the formation of the dotted structure, thus leading to the low activity of PCMS-0.5 for PHE. The high activity of PCMS-1 than the rods-like ZnO/ZnS composite has also supported the conclusion. Notably, although the higher S_BET_ of PCMS-2.5 (265 m^2^/g), its activity is lower than that of PCMS-1, which may be related with the lower amount of ZnO in former than later. Also, the normalized specific area activity is about 1.88, 1.78, 1.17 and 1.72 μmol g. h^−1^ m^−2^ for PCMS-1, PCMS-0.5, PCMS-2.5, PCMS-5. The PCMS-1 has also exhibited a highest normalized specific area activity in PHE. Therefore, the cooperative action of moderate (higher) S_BET_ surface, ZnO content and the (smaller) crystalline size makes the high activity of PCMS-1 sample among all PCMS samples. The formation of uniform, ZnO-dotted structure is a key to give high PHE activity. In reuse test (Figure 4b), although the rate of hydrogen evolution is slightly decreased at initial 1 h in 2-5nd cycles, the final amount of hydrogen after 3 h reaction is similar for different cycles. The apparent quantum efficiency (QE) of PCMS-1 is about 34.83% at 313 nm, 12.16% at 365 nm and 0.86% at 405 nm. The results indicate the high activity and well reuse ability of PCMS-1 for PHE. Also, we can see that the PCMS-1 show high QE when the irradiated wavelength is close to the intrinsic irradiated wavelength of ZnS. The deviation of irradiated wavelength from the intrinsic irradiated wavelength of ZnS can result the obvious decrease of apparent QE. The results imply that the ZnS is the light absorber. In addition, according to the energy-band structure of ZnS and ZnO, the effective photocatalytic hydrogen evolution can be realized only in the case of ZnS as the primary light absorber and the dotted ZnO as the co-catalyst (the formation of Type II photocatalyst^42^).

### The Origin of Active Catalytic Sites for H_2_ Evolution

**Figure 5:**
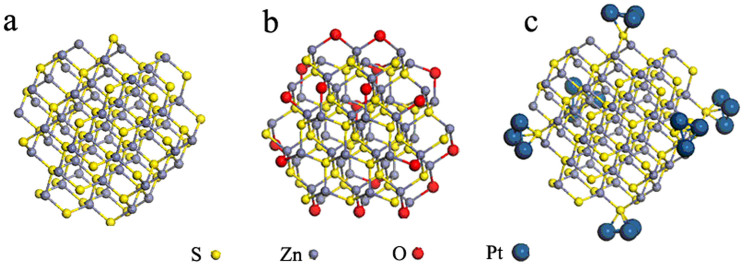
the model of ZnS, ZnO-dotted ZnS and Pt modified ZnS clusters.

The free-Pt, high efficient PHE of PCMS is interesting. Generally, the Pt is needed to provide the active catalytic sites for PHE. In PCMS-1 system, no Pt is used in PHE test, implying the presence of active catalytic site in PCMS itself. What and where is the active catalytic site? To understand the origin deeply, the theoretical calculation is first performed within density functional theory. The corresponding models of ZnS, ZnO-dotted ZnS and Pt-modified ZnS are shown in Figure 5.

**Figure 6:**
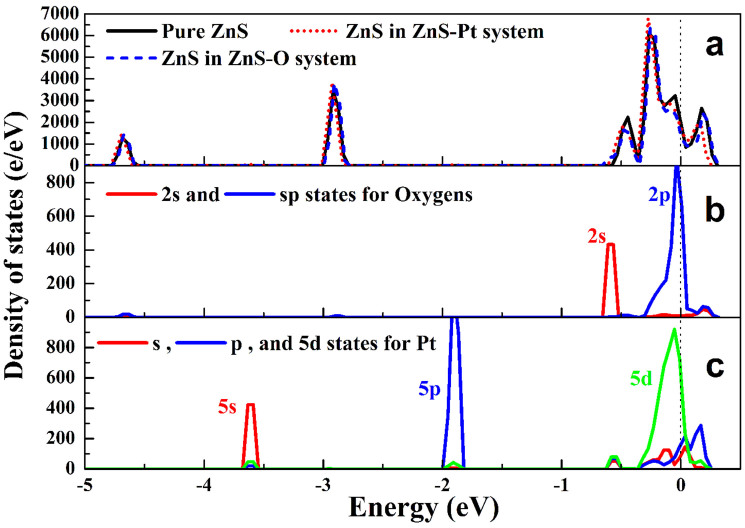
(a) Total and (b, c) partial density of states (DOSs) of ZnS, Pt modified ZnS (ZnS-Pt), and ZnO-dotted ZnS (ZnS-O) systems.

Figure 6 shows the total and partial density of states (DOSs) of pure ZnS clusters, Pt modified ZnS (ZnS-Pt), and ZnO-dotted ZnS (ZnS-O) systems. The results indicate that the total DOSs of ZnS-Pt and ZnS-O systems near the Fermi energy (0.0 eV) both shift to lower energy position (Figure 6a). According to the partial DOSs, it can be found that O2p states in ZnS-O system near the Fermi level (Figure 6b) are partially filled, while Pt5d states in ZnS-Pt system (Figure 6c) also exhibits similar characteristics. The calculation indicates that the dotting of ZnO on ZnS results in the shift of the charge from ZnS to ZnO, being similar to that of Pt-modification. It has been reported that the MoS_2_ can be used as like-Pt co-catalyst for Pt-free PHE. In the PCMS, the dotting of ZnO on ZnS will result in the formation of S-Zn-O surface sites (Figure 5b and Figure 1b). Therefore, on the basic of the theoretical calculation, we have proposed that the ZnO-dotted ZnS should have similar properties with ZnS decorated with Pt, which should be important reason for Pt-free PHE of PCMS.

**Figure 7:**
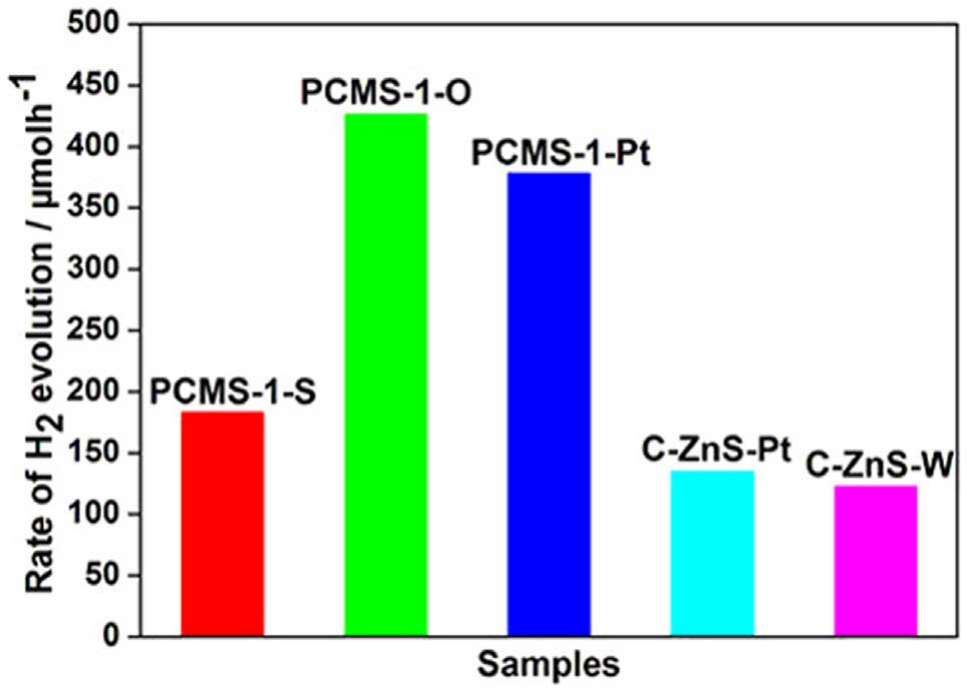
The PHE activity of control samples: PCMS-1-S (prepared by treatment of PCMS-1 under H_2_S atmosphere), PCMS-1-O (prepared by the treatment of PCMS-1 under air), PCMS-1-Pt (testing PHE activity of PCMS-1 in the presence of 1.wt% Pt) and C-ZnS-Pt (testing PHE activity of C-ZnS in the presence of Pt), C-ZnS-W (prepared by the treatment of C-ZnS at 120°C for 4 h).

Several experiments are designed to further verify that the ZnO dotting is intrinsic origin for the formation of the active catalytic sites for H_2_ evolution. Firstly, the PCMS-1 is calcinated under air at 200°C for 2 h. The treatment can lead to the increase of ZnO amount in the sample, and as-prepared sample is marked as PCMS-1-O. The treatment of PCMS-1 under N_2_ by using thiourea as S source at 200°C for 2 h results in the formation of PCMS-1-S sample. Under the condition, the H_2_S from the decomposition of thiourea can react with PCMS-1, resulting in the decrease of ZnO amount in the sample. TEM and XRD tests indicate the same structure of PCMS-1-O, PCMS-1-S with PCMS-1 (Figure S10, S11). However, no vibration of Zn-O is found in Raman spectrum of PCMS-1-S, implying a lower ZnO content than that in PCMS-1 (Figure S12). In contrast, the PCMS-1-O gives the enhanced peaks of Zn-O vibration (Figure S12), indicating the increase of ZnO amount after the PCMS-1 is treated in air. It is speculated that the PCMS-1-O would give enhanced PHE activity, and PCMS-1-S has contrary change based on the role of ZnO dotting speculated above. Indeed, the tests indicate that the H_2_-production rate is about 427 μmol h^−1^ for PCMS-1-O, which is much higher than 367 μmol h^−1^ for PCMS-1 (Figure 7). However, the H_2_-production rate of PCMS-1-S is 183 μmol h^−1^, much lower than that of PCMS-1 (Figure 7). The results verify the active catalytic sites for hydrogen production is from ZnO dotting.

Further test is to perform the PHE test with the assistant of Pt. Generally, the Pt can provide effective proton reductions sites, hence enhances the H_2_ production activity for PHE. Notably, the PHE activity of PCMS-1 has no obvious change with the addition of Pt (1 wt.%) in PHE test (the PHE activity is changed from 367 μmol h^−1^ to 378 μmol h^−1^) (Figure 7). The result implies the presence of plentiful catalytic sites for hydrogen production in PCMS-1. So, the addition of Pt can not give obvious influence of the PHE activity of PCMS-1 sample. From Figure 7, we can see that the C-ZnS only give a low PHE activity of 17 μmol h^−1^. It is proposed that supplying additional active catalytic sites would enhance the PHE activity of C-ZnS. Two methods are used to realize the aim: the dotting of ZnO on C-ZnS and adding Pt. After addition of Pt (1 wt.%) in the test, the PHE activity of C-ZnS can be enhanced from 17 μmol h^−1^ to 135 μmol h^−1^. A hydrothermal treatment of C-ZnS is used to dot the ZnO on C-ZnS (the sample is denoted as C-ZnS-W). Interestingly, the C-ZnS-W gives an activity of 122 μmol h^−1^ under Pt-free condition, which is comparable with C-ZnS-Pt. The dotting of ZnO on ZnS will result in the formation of S-Zn-O surface sites (Figure 5b). The tests verify that the dotting of ZnO on ZnS (formation of S-Zn-O surface sites) can result in the formation of active catalytic sites for hydrogen production.

The work function (WF) is measured to give further understanding on the PHE performance of the samples. The SKP measurement is was performed to measure the WF. Three samples, ZnO (PCMS-0), PCMS-0.5 (the mixed phase of ZnO and ZnS) and PCMS-1 (ZnO-dotted ZnS), were coated on FTO to form a thin film. The WF of the commercial Pt black was also test. The test indicated that the WF of ZnO, PCMS-0.5 and PCMS-1 is about 5.17, 5.20 and 5.35 eV, respectively, while that of Pt black is about 5.36 eV. The higher WF (close to Pt) of PCMS-1 implies that S-Zn-O surface sites formed by ZnO dotting have strong ability for trapping the electrons and the trapped active electrons can reduce H^+^ to H_2_ on the S-Zn-O sites. The test of WF further supports the conclusion that the S-Zn-O surface sites can be active sites for H_2_ production due to their similar WF with Pt metal. The photoelectrochemical test implies that PCMS-1 have the fast electron mobility, high carrier density and positive potential for the evolution of H_2_, which should be relative with excellent PHE activity of PCMS sample (Figure S13 and S14, ESI). We can see that the overpotential (about −0.5 V vs RHE ) is more negative than that of Pt (about 0 V vs. RHE). This means that electrocatalytic ability of PCMS-1 for the evolution of H_2_ is not as strong as metal Pt. Nevertheless, the overpotentials of the PCMS-1 are very close with that of the photocatalytic systems containing MoS_2_^19^ those are well-known cocatalyst for PHE, but with a lower current density than MoS_2_ and WS_2_. Generally, the films for electrochemical test need to be treated at higher temperature (typically, 400°C) to make the film firmly on the FTO substrate. We considered that such a higher temperature will lead to the change of PCMS-1 (the oxidation and/or structure change). To avoid this, the PCMS-1 film was treated at lower temperature (100°C) for 6 h under air. The low temperature may lead the film not firmly enough which could cause high interface resistance between FTO and semiconductor film, thus lead to low photocurrent. Therefore, the lower photocurrent of PCMS-1 film should be relative with low heating-treatment temperature. The results further support that the PCMS-1 have the ability to decrease the overpotential for the hydrogen evolution and to catalyze the hydrogen evolution. Therefore, through the combination of the theoretical calculation and serials of designed experiments, we demonstrate firstly that ZnO dotting can results in the formation of the active catalytic sites for PHE, just like the Pt modification, which should be origin of high-efficient, Pt-free PHE of PCMS samples.

In summary, we have demonstrated that ZnO-dotted porous ZnS cluster microsphere can be used as high efficient catalyst for Pt-free PHE. By combination of a series of the experiments and theoretical calculation, the double roles of ZnO dotting were demonstrated firstly, that is, the ZnO dotting not only makes the photo-generated e^−^/h^+^ separate efficiently, but also results in the formation of the active catalytic sites for hydrogen production. The high-efficient, free-Pt PHE are intimately relative with the porous structure and small crystalline size for promoting the effective separation of the photogenerated e^−^/h^+^ pairs, as well as the formation of active catalytic sites for hydrogen production after ZnO dotting. The easy handle process, low preparation cost and good PHE activity with no need of noble Pt make the large potential of PCMS for PHE.

## Methods

### The preparation of PCMS

The PCMS were prepared via a simple solvothermal method. For the synthesis of PCMS-1, 1 mmol of zinc acetate and 1 mmol of thiourea were added into 80 mL of ethylene glycol to form a clear solution after stirring for 1 hour at room temperature. The solution was transferred into an autoclave with an inner Teflon lining of 100 mL. The autoclave was maintained at 180°C for 12 hours and then air-cooled to room temperature. The product was collected by centrifugation, washed several times with distilled water and ethanol and finally air dried in an oven at 60°C for 10 h. The sample was denoted as PCMS-1, where 1 represented the molar ratio (MR) of thiourea and zinc acetate. The samples prepared with different ratios of thiourea and zinc acetate (1/2, 2.5/1, 5/1) under other identical conditions were denoted as PCMS-0.5, PCMS-2.5 and PCMS-5 respectively. The commercial ZnS (C-ZnS) was also used as reference.

### Characterizations

The structure was observed by transmission electron microscopy (TEM: JEM-2100) operated at 200 kV. X-ray powder diffraction (XRD) test was performed on a Bruker D8 diffractometer with monochromatized Cu K radiation with accelerating voltage 40 kV and the applied current 20 mA. Fourier transform infrared spectrum (FTIR) was collected using in a NICOLETiS10. Raman measurements were performed with a Jobin Yvon HR 800 micro-Raman spectrometer at 457.9 nm. The nitrogen adsorption/desorption isotherms were measured at 77 K by using a Micromeritics Tristar II. The specific surface area of the materials was calculated by the Brunauer–Emmett–Teller (BET) method. X-ray photoelectron spectroscopy (XPS) analysis was performed with VG ESCALAB MK II using an MgKa (1253.6 eV) achromatic X-ray radiation. The scanning Kelvin probe (SKP, SKP5050 system, Scotland) measurement was performed at normal conditions of laboratory in ambient atmosphere. A gold electrode was used as the reference electrode. The value of work function is obtained by averaging the value acquired from 49 points. Photoelectrochemical measurements were performed using a Princeton Versa STAT 3 in a standard three-compartment cell consisting of a working electrode, a Pt slice counter electrode, and a saturated Ag/AgCl reference electrode. For SKP and electrochemical test, the samples were spread on FTO to form a film. The film for electrochemical test was treated at lower temperature (100°C) for 6 h under air to make the film firmly. To protect the PCMS from oxidation, the heating temperature is much lower than that in literature (about 400°C). The X-ray absorption edge spectroscopy (XAES) data were measured at room temperature in transmission mode at beam line BL14W1 of Shanghai Synchrotron Radiation Facility (SSRF), China.

### Photocatalytic H_2_ production

The photocatalytic H_2_ evolution from water was conducted in an online photocatalytic hydrogen production system (AuLight, Beijing, CEL-SPH2N). The photocatalyst powders (0.04 g) were dispersed by the ultrasonication in an aqueous solution (100 mL) containing 0.1 M Na_2_S and 0.05 M Na_2_SO_3_ as electron donors. The reaction was carried out by irradiating the suspension with UV light from a 300 W Xe lamp with a 200–400 nm reflection filter, which means the wavelength of light is approximately 200–400 nm. The lamp power incident on the surface of the reaction solution is 200 mW/cm^2^. Prior to the irradiation, the system was deaerated by evacuation to remove the air inside in order to assure anaerobic conditions in the reaction system. Gas evolution was observed only under photo-irradiation, and was analyzed by an on-line gas chromatograph (SP7800, TCD, molecular sieve 5Å, N_2_ carrier, Beijing Keruida Limited).

The most of PHE tests were performed under free-Pt condition. In the case of adding Pt as co-catalyst, the samples were marked as sample-Pt. For example, the PCMS-1-Pt indicates that the PHE test was performed by using PCMS-1 as catalyst with the assistance of Pt.

Several experiments were designed to elucidate the origin of active catalytic sites. The samples include PCMS-1-O, PCMS-1-S, PCMS-1-Pt, C-ZnS-W, C-ZnS-Pt. The corresponding preparation (use) conditions of the samples are as follows:

The PCMS-1-O was obtained by the calcination of PCMS-1 under air at 200°C for 2 h. The treatment can lead to the increase of the dotted ZnO amount.

The PCMS-1-S was obtained by the treatment of PCMS-1 under N_2_ in the presence of thiourea as S source at 200°C for 2 h. Under the condition, the H_2_S from the decomposition of thiourea can react with PCMS-1, thus resulting in the decrease of the dotted ZnO amount in the sample.

PCMS-1-Pt indicated that the PHE test was performed by using PCMS-1 as catalyst with the assistance of Pt co-catalyst (1 wt.%).

C-ZnS-Pt indicates that the PHE test was performed by using C-ZnS as catalyst with the assistance of Pt co-catalyst.

C-ZnS-W sample was obtained by the hydrothermal treatment of C-ZnS at 120°C for 4 h. The treatment can lead to the formation of a little of the dotted ZnO in C-ZnS.

### Theoretical calculation

The calculations were performed within the density functional theory (DFT)^43^ framework embedded in the Dmol3 code^44^. The exchange-correlation energy was treated by the PW91 functional^45^ in generalized gradient approximation (GGA) form. The basis functions were described by a double numerical plus polarized (DNP) basis set, and the global orbital cutoff was set to 4.0 Å. To obtain a stable structure, the geometry optimization procedure was repeated until the average force on the atoms was less than 0.05 eV/Å and the energy change less than 1.0 × 10^−5^ eV/atom.

## Author Contributions

A.P.W. performed synthesis experiments and carried out photocatalytic evaluation. C.G.T. and H.G.F. designed the experiment. L.Q.J. and Y.Q. contributed in the analysis of photo-electrochemical test. Y.X. performed the theoretical calculation. J.Q.W. and B.J.J contributed in the test and analysis of the X-ray absorption edge spectroscopy. A.P.Wu, C.G.T. and H.G.F. wrote the manuscript. All authors reviewed the manuscript.

## Supplementary Material

Supplementary Information Supplementary Information

## Figures and Tables

**Table 1:** Fit parameters of Standard ZnS, PCMS-0.5, PCMS-1, PCMS-2.5 and PCMS-5 samples

Sample	shell	*N*	*R*(Å)	σ^2^(×10^-3^ Å^2^)	Δ*E*_0_(eV)
Standard ZnS	Zn-S	4	2.35 ± 0.02	4.6 ± 0.3	4.6
	Zn-S-Zn	12	3.85 ± 0.02	15.3 ± 0.9	5.1
	Zn-S	9	4.55 ± 0.02	12.5 ± 1.9	8.5
PCMS-0.5	Zn-S	2.3	2.32 ± 0.02	6.9 ± 2.2	3.0
	Zn-O	1.7	1.99 ± 0.03	6.5 ± 4.0	3.0
PCMS-1	Zn-S	3.5	2.33 ± 0.02	5.2 ± 0.5	3.7
	Zn-O	1	1.97 ± 0.02	6.9 ± 3.2	3.7
	Zn-S-Zn	4.5	3.84 ± 0.04	15.9 ± 3.3	5.1
	Zn-S	3	4.51 ± 0.10	15.5 ± 9.6	8.5
PCMS-2.5	Zn-S	4	2.34 ± 0.02	5.4 ± 0.6	4.6
	Zn-S-Zn	5	3.83 ± 0.03	15.8 ± 4.1	5.1
	Zn-S	3	4.52 ± 0.07	14.8 ± 12.3	8.5
PCMS-5	Zn-S	4	2.33 ± 0.02	5.1 ± 0.5	3.7
	Zn-S-Zn	6	3.82 ± 0.02	16.1 ± 2.8	5.1
	Zn-S	4	4.55 ± 0.05	15.1 ± 7.8	8.5

Coordination number, *N*; bond distance, *R*; Debye-waller factor, σ^2^; inner potential shift, Δ*E*_0_.
