# Supplementary material for: ZnO-dotted porous ZnS cluster microspheres for high efficient, Pt-free photocatalytic hydrogen evolution
Source: Sci Rep. 2015 Mar 9;5:8858. doi: 10.1038/srep08858 (PMC4352920; doi:10.1038/srep08858)
Supplement: Supplementary Information [file srep08858-s1.doc]

**Supporting Information**

ZnO-dotted porous ZnS cluster microspheres for high efficient, Pt-free photocatalytic hydrogen evolution

Aiping Wu,^a^ Liqiang Jing,^a^ Jianqiang Wang,^b^ Yang Qu,^a^ Ying Xie,^a^ Baojiang Jiang,^a^ Chungui Tian^a^* and Honggang Fu^a^*

a Key Laboratory of Functional Inorganic Material Chemistry, Ministry of Education of the People’s Republic of China, Heilongjiang University, Harbin 150080 (P. R. China),Fax: (+ 86) 451-8666-1259
E-mail: fuhg@vip.sina.com; chunguitianhq@163.com

b Shanghai Synchrotron Radiation Facility (SSRF), Shanghai Institute of Applied Physics, Chinese Academy of Sciences, Shanghai 201204, China


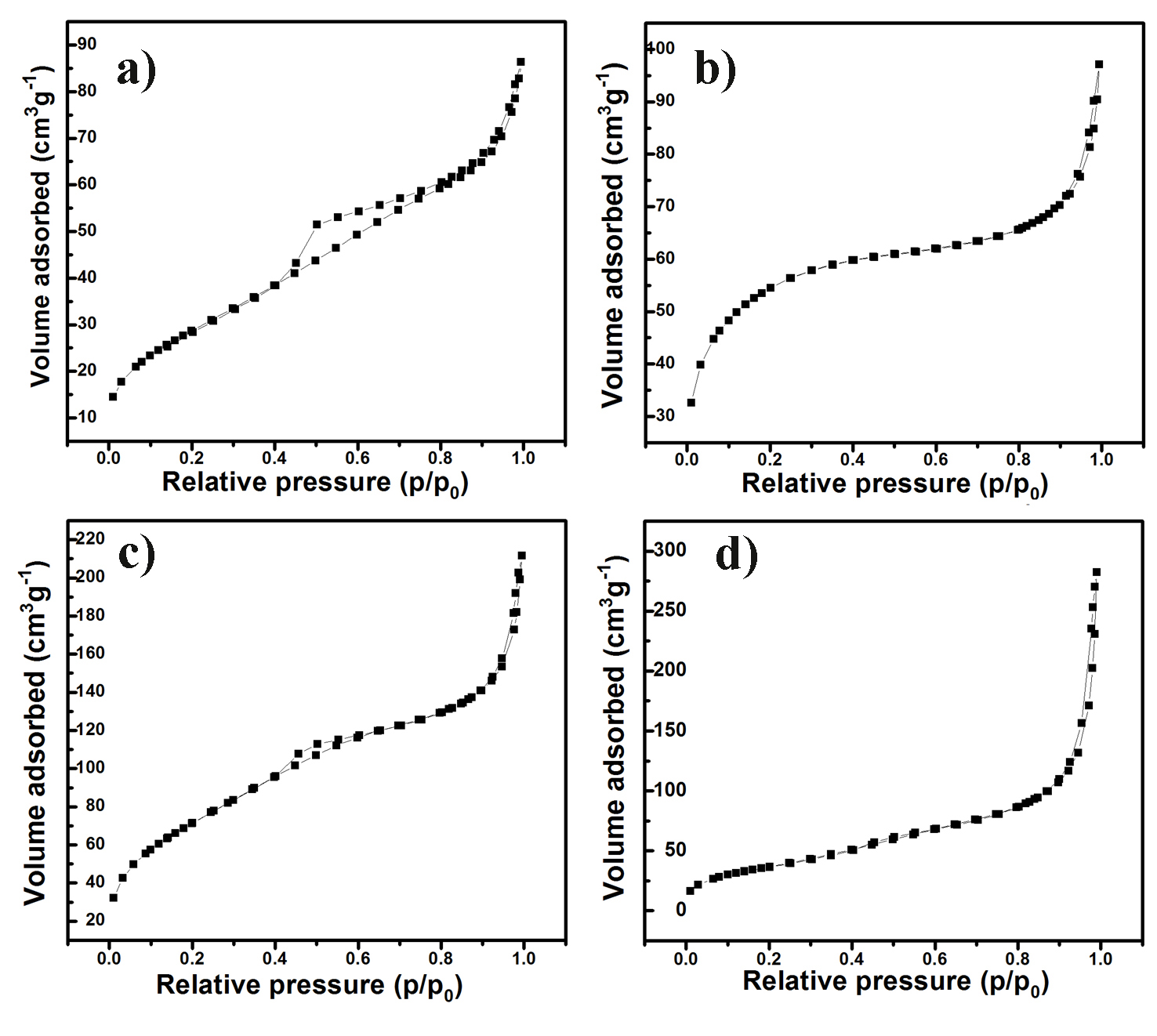


**Fig. S1** N_2_ adsorption–desorption isotherms at -196 ^o^C of a) PCMS-0.5, b) PCMS-1, c) PCMS-2.5, and d) PCMS-5.

Table S1. S_BET_ of PCMS samples


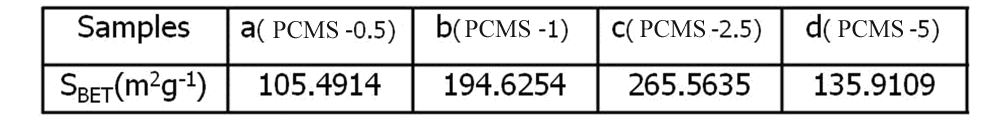


.
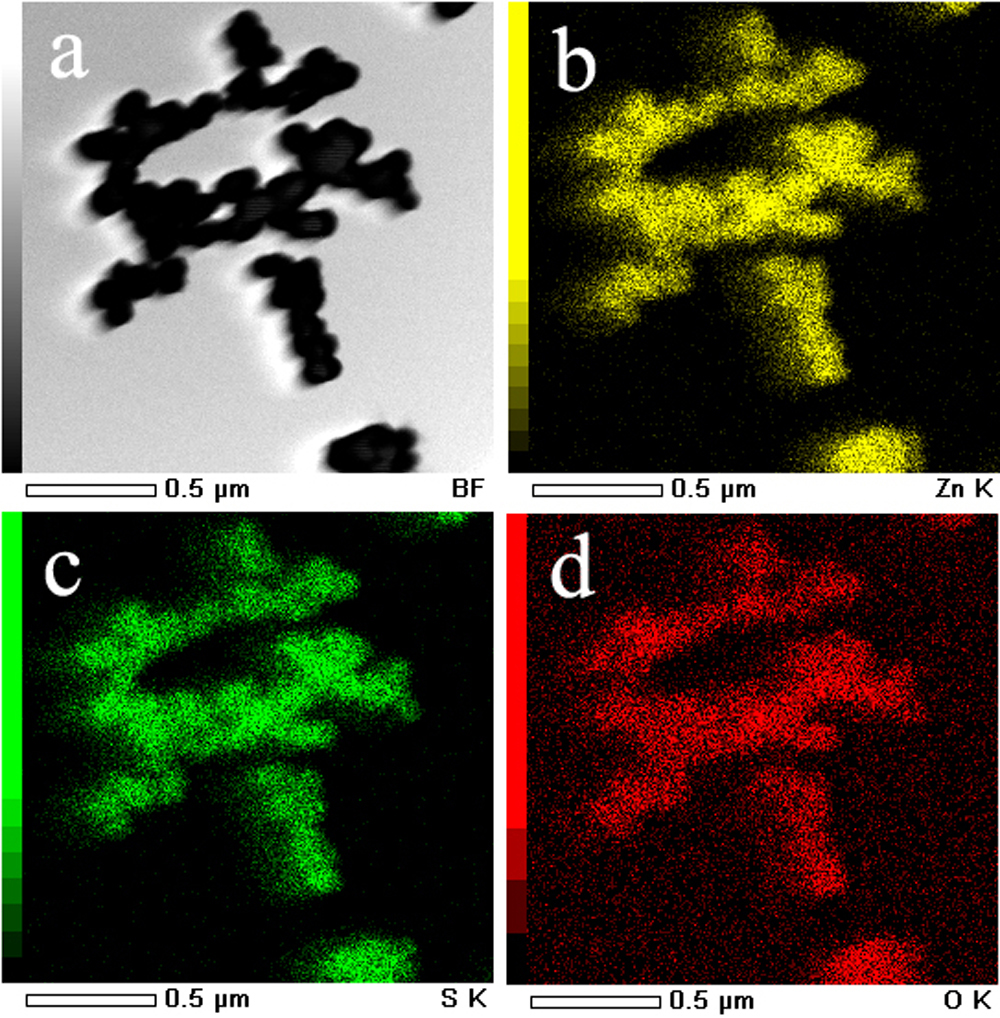


**Fig. S2** a) BF-STEM images of PCMS-1and the (b-d) is corresponding elemental mapping b) Zn, c) S and d) O. The test indicates the existence of O, S and Zn in PCMS-1, and the homogeneous distribution of Zn, S and O elements throughout the microsphere, implying the presence of ZnO in the PCMS homogeneously.


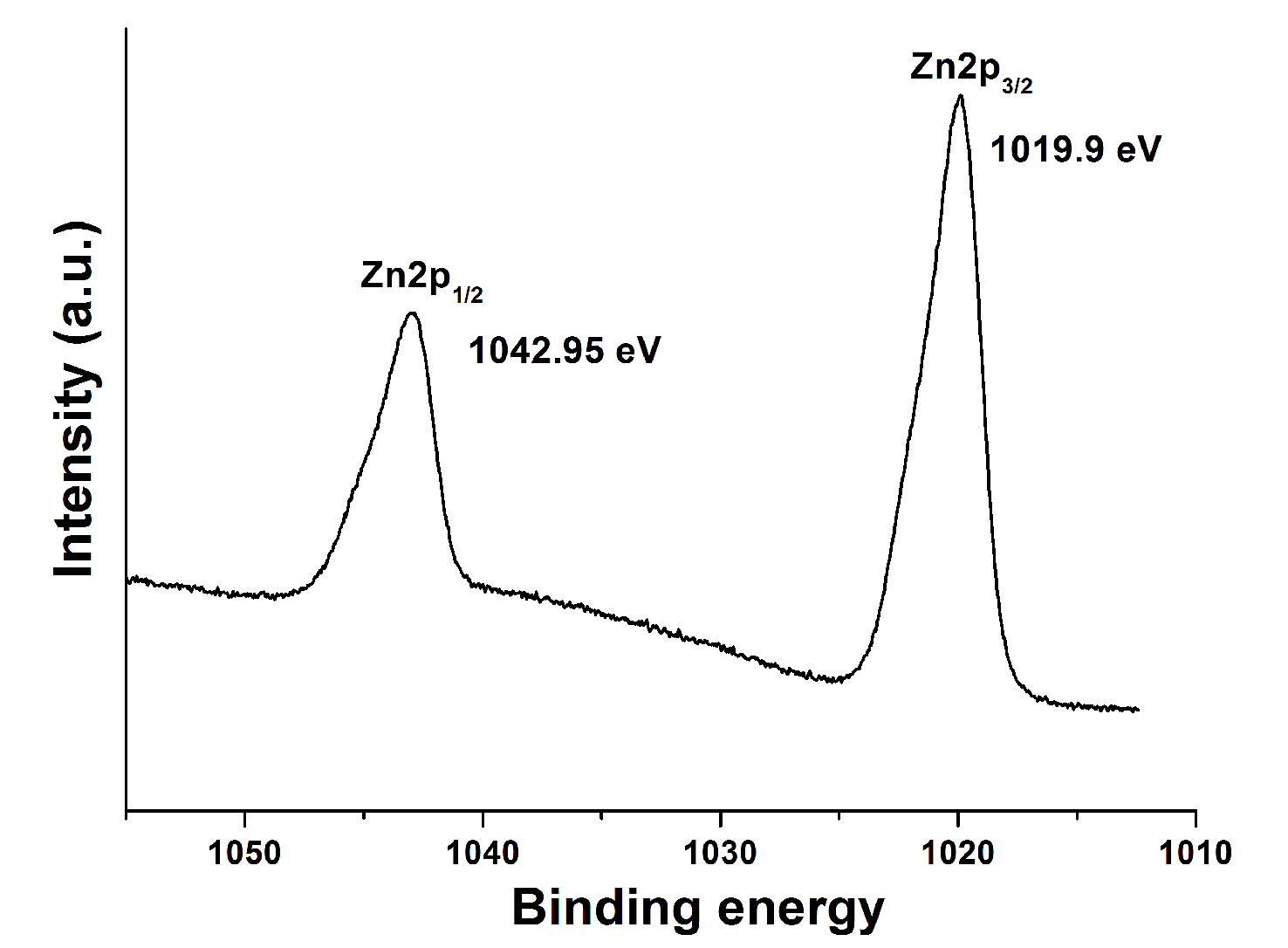


**Fig. S3** the Zn2p XPS spectrum of PCMS-1.

The high resolution XPS spectrum in Fig. 3 indicates that Zn 2p1/2 and 2p3/2 are located at 1042.95eV and 1019.9 eV, which are characteristics for Zn^2+^ in ZnS [Ref. 1]. No other peaks can be decoverluted, implying that no Zn^0^ in the sample.

**Ref. 1:** M. H. Ullah, B. Chon, T. Joob, M. Sona, Il Kima, C.-S. Ha, Journal of Colloid and Interface Science, 2007, 316, 939–946.


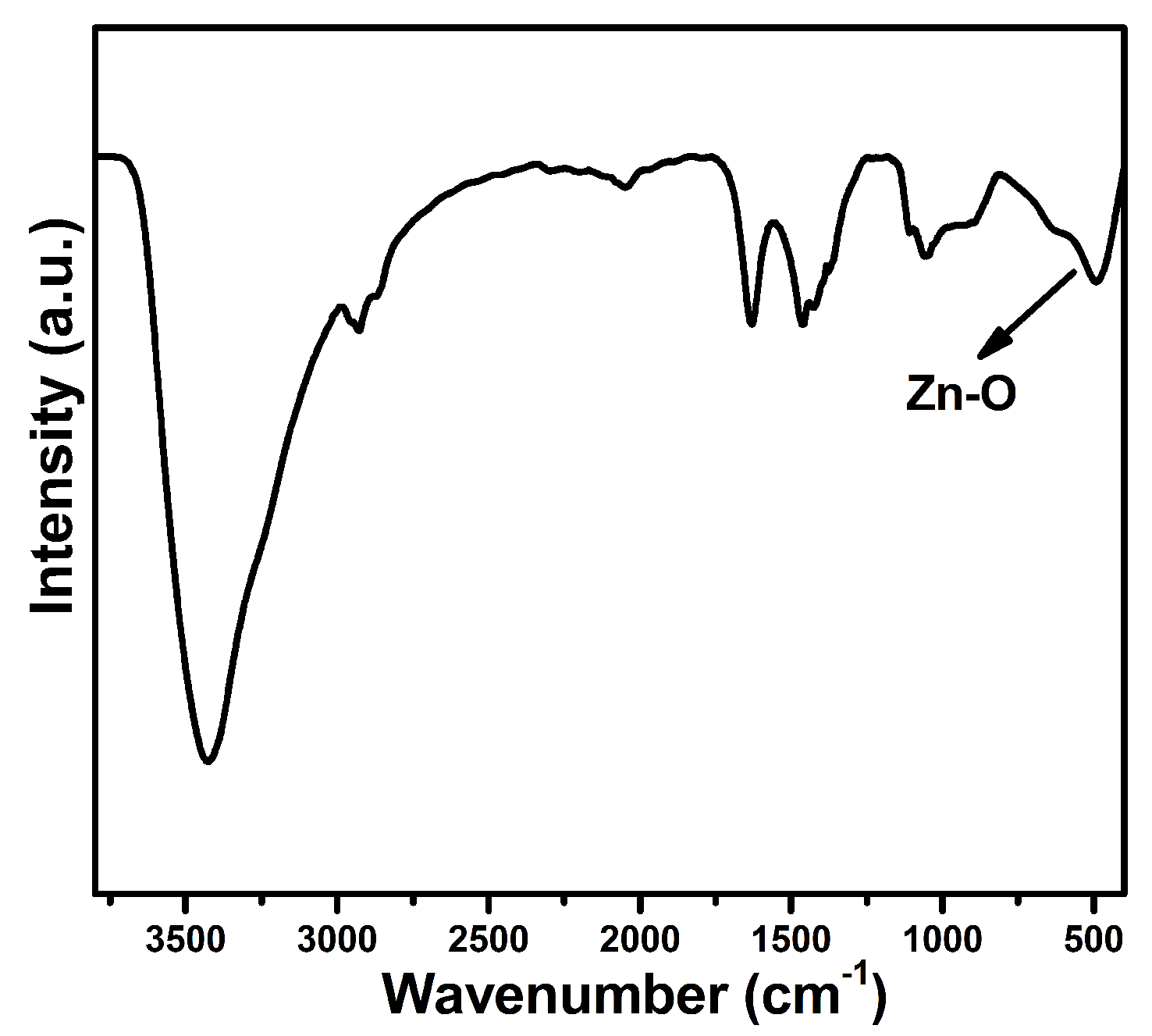


**Fig. S4** IR spectrum of PCMS-1. The typical vibration of Zn–O bond for ZnO can be seen at about 500 cm^-1^ with noticeable intensity.


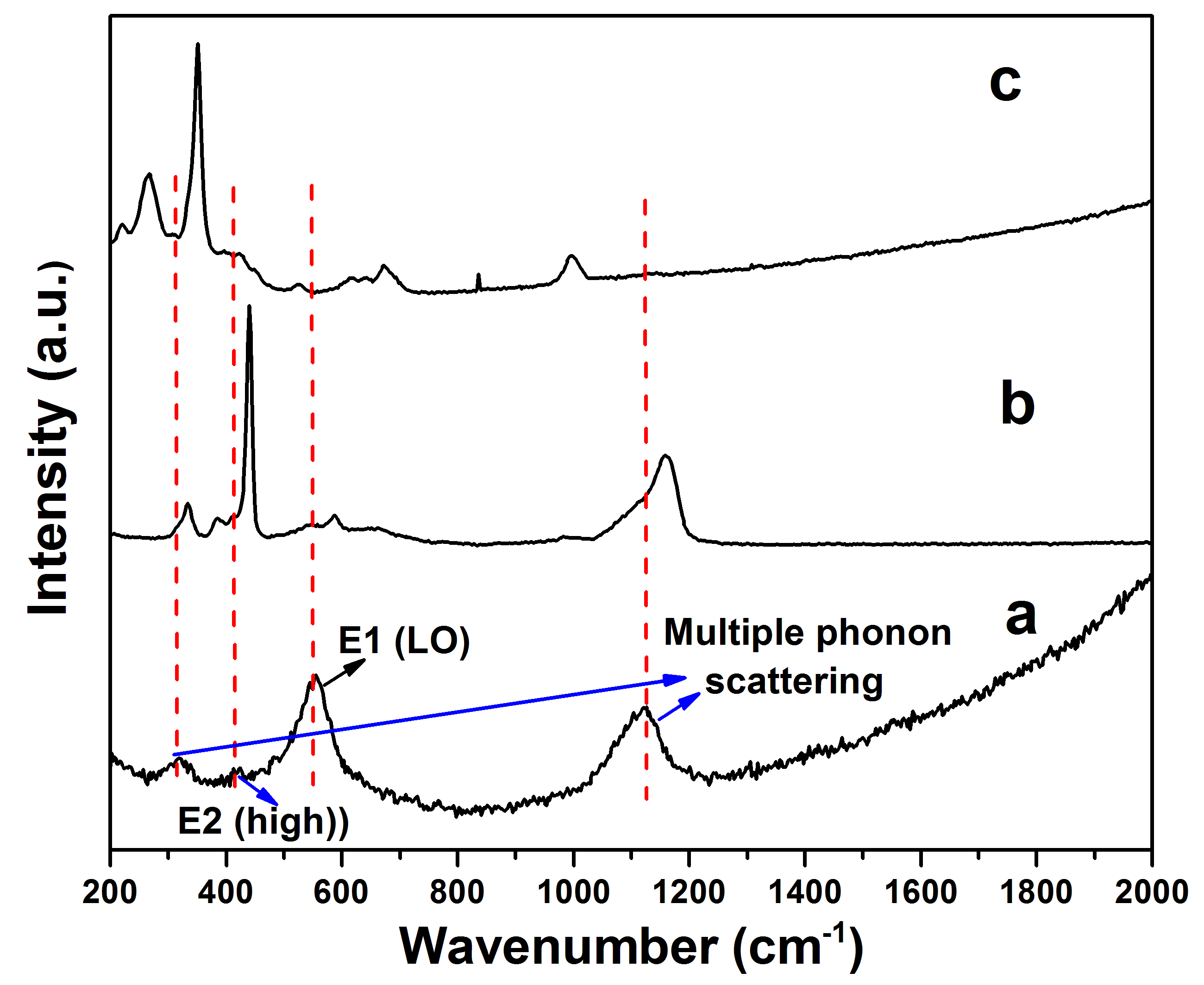


**Fig. S5** Raman spectra of a) PCMS-1, b) c-ZnO and c) c-ZnS.

We can see that the peaks of PCMS-1 show good consistence with that of commercial ZnO (C-ZnO). Nevertheless, in comparison with the C-ZnO, we still can observe the some shift of peak position and change of relative intensity in the Raman spectra of PCMS-1 sample. The shift and change should be relative with the lower coordination number of Zn-O in PCMS-1 than that in C-ZnO.

The peaks in Raman spectrum of PCMS-1 are not ascribed in the ZnS based on the followed points: a) By the comparison of Raman spectra, we can find the less consistence between the C-ZnS and PCMS-1 in many characteristic peaks; b) After heating PCMS-1 in air (the treatment can result in the enhancement of Zn-O in the PCMS-1), the Raman peaks can be further enhanced with no change in peak position, implying the peaks are from the vibration of Zn-O; c) With the enhancement of ZnS by heating at H_2_S atmosphere, the Raman peaks in original PCMS-1 decrease dramatically (almost disappeared). The results further support that the peaks in original PCMS-1 are from the vibration of ZnO dotting. Notably, although XRD, TEM and EXAFS demonstrated that the ZnS as main component of PCMS, we can not observed the peaks corresponding to ZnS, even in the samples with low content of dotted ZnO (Fig .S7 below). This may be due to the presence of some organic residues or small size of ZnS crystalline grain..


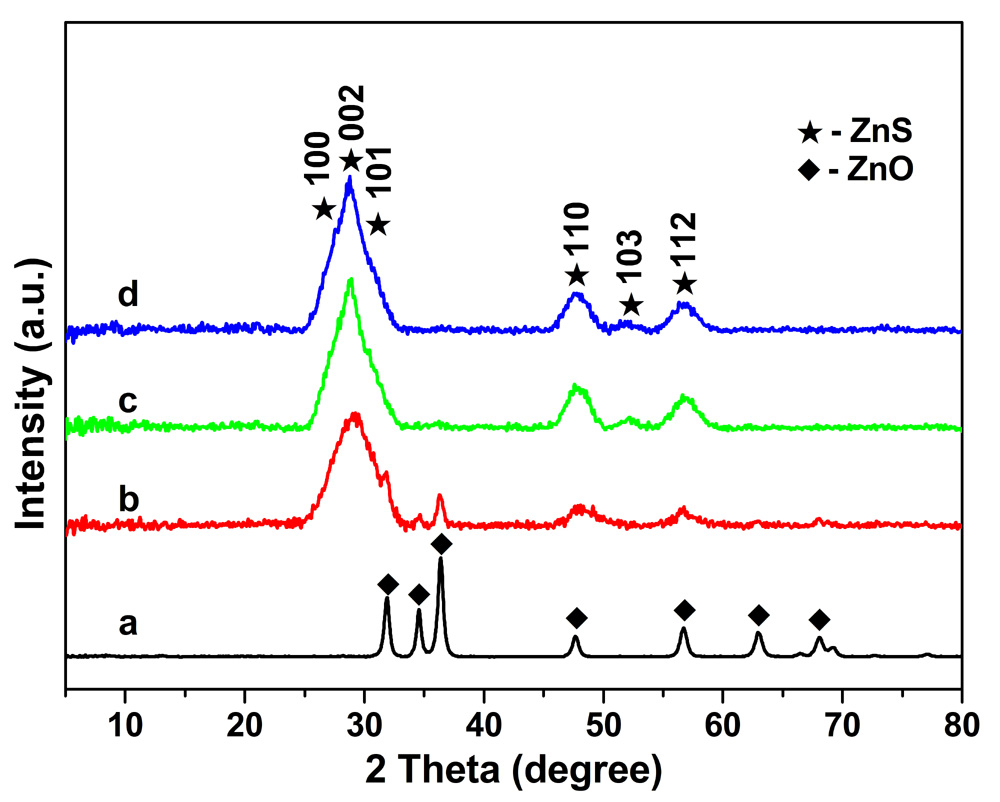


**Fig. S6** XRD patterns of (a) PCMS-0 (ZnO), (b) PCMS-0.5, (c) PCMS -2.5 and (e) PCMS -5.

For PCMS-0, the intensive peaks located at 2θ = 31.7°, 34.4°, 36.2°, 47.5°, 56.6°, 62.8°, and 67.8° can be indexed to (100), (002), (101), (102), (110), (103) and (112) diffractions of hexagonal structure ZnO (wurtzite) (JCPDS card no. 36-1451). No other peaks can be observed. Both diffraction peaks belonging to ZnO and ZnS can be found in PCMS-0.5 sample, indicating the co-existence of ZnS and ZnO. The peaks belonging to ZnO is lower than that of ZnS, implying the relative low content of ZnO in the sample. For PCMS-2.5 and PCMS-5, no obvious peaks of ZnO can be observed, besides the intensive peaks belonging ZnS, indicating the more low amount of ZnO in the samples.


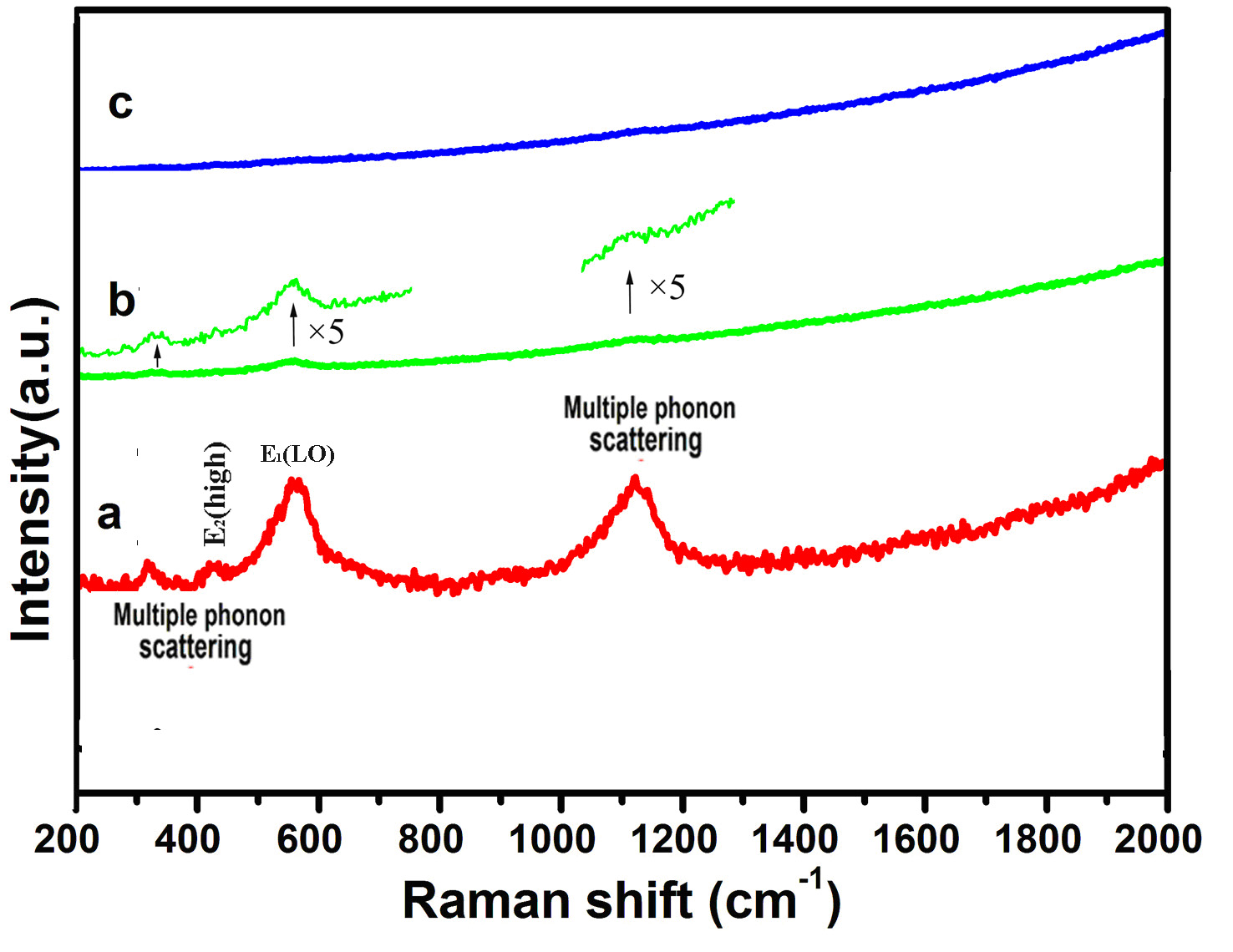


**Fig. S7** Raman spectra of (a) PCMS-0.5, (b) PCMS-2.5, (c) PCMS-5.

The peaks belonging to ZnO can be seen in Raman spectrum PCMS-0.5 and PCMS-2.5. We can see that the peak intensity decrease gradually in the order of PCMS-0.5>PCMS-2.5> PCMS-5. For PCMS-5, no obvious peaks of Zn-O vibration can be found. The results indicate the decrease of ZnO content with the increase of MR value in the synthesis of PCMS samples.


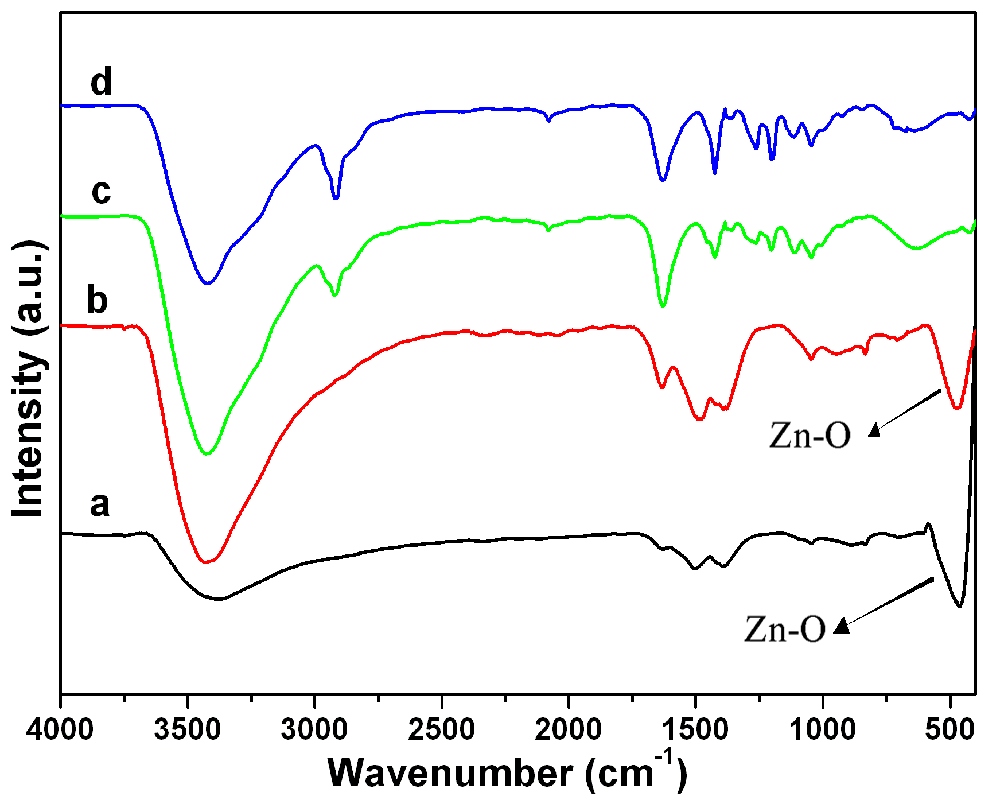


**Fig. S8** IR spectra of a) PCMS-0 (ZnO), b) PCMS-0.5, c) PCMS-2.5 and (d) PCMS-5.

The peak corresponding to Zn-O vibration with notable intensity can be found in PCMS-0 and PCMS-0.5 samples. While for PCMS-2.5 and PCMS-5, no obvious peaks about Zn-O vibration can be observed, implying the very lower content of ZnO in the samples. The results indicate the decrease of ZnO amount with the increase of MR values.


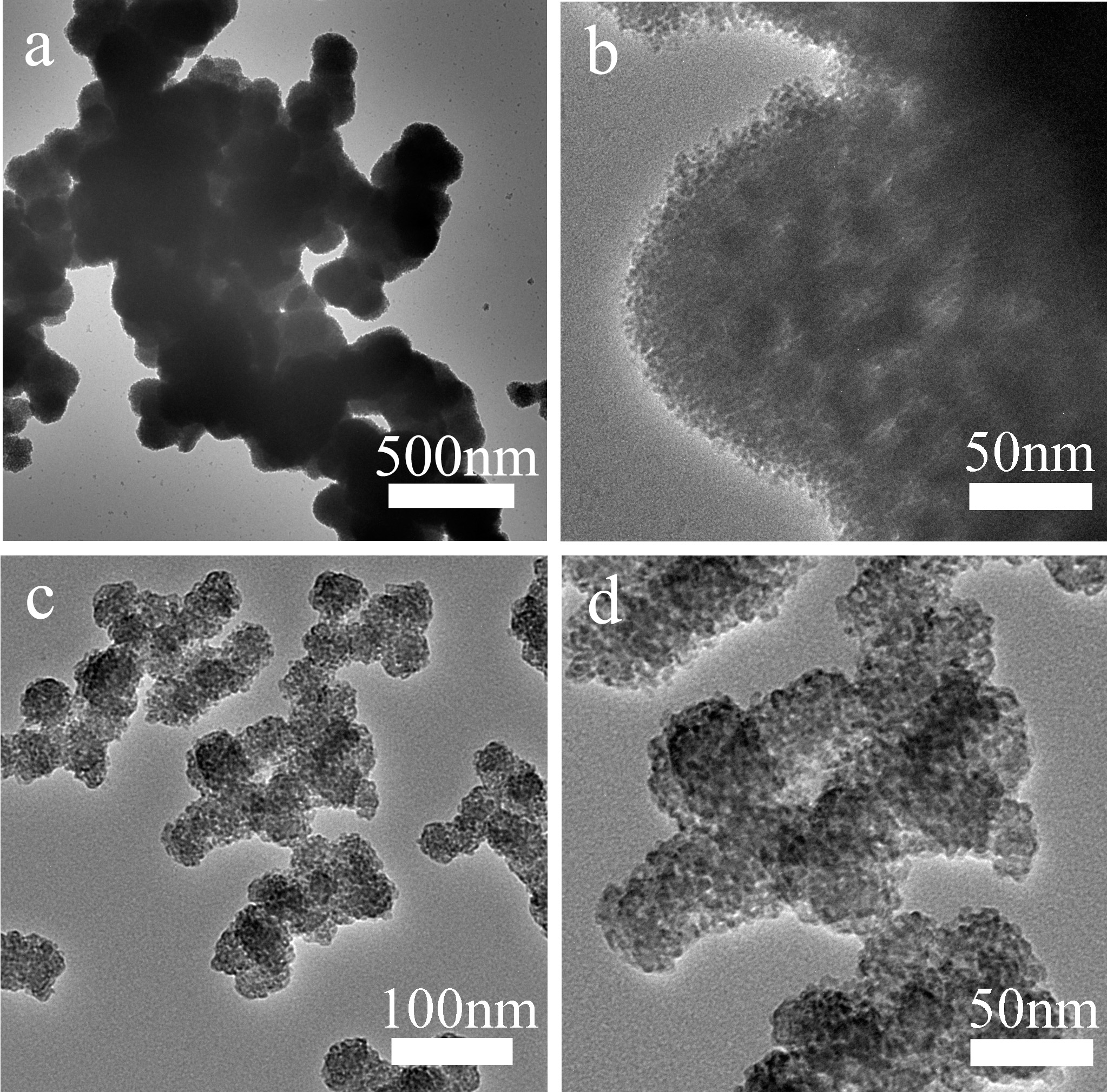


**Fig. S9** TEM images of (a,b) PCMS-0.5 and (c, d) PCMS-5.


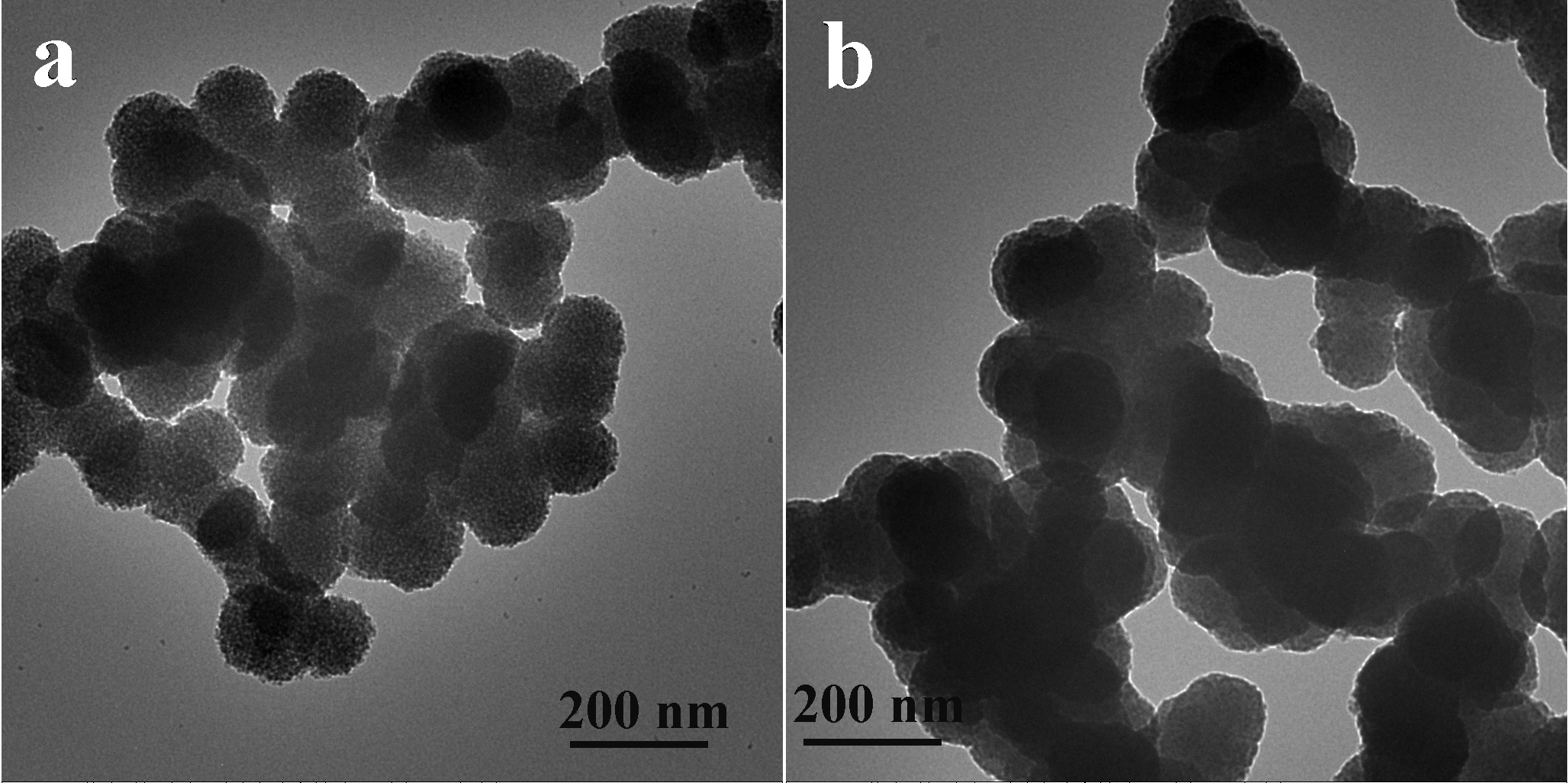


**Fig. S10** TEM images of (a) PCMS -1-O and (b) PCMS -1-S.


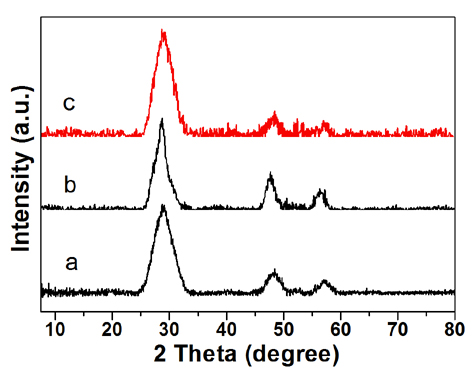


**Fig. S11** XRD patterns of (a) PCMS-1, (b) PCMS -1-S and (c) PCMS -1-O.

**Fig. S10 and S11** shows TEM images and XRD patterns of PCMS-1-S, PCMS-1-O. The PCMS-1-S was obtained by treatment of PCMS-1 under N_2_ in the presence of thiourea at 200 ^o^C for 2h. Under the condition, the thiourea can decompose to release H_2_S. So, this treatment will result the decrease of ZnO amount in the sample. The PCMS-1-O is from the calcination of PCMS-1 under air at 200 ℃ for 2 h. This treatment can lead the further replacement of S by O, thus give increased amount of ZnO components in the PCMS-1-O. Based on TEM test (Fig. S10), we can observe the same size and morphology of PCMS-1-O, PCMS-1-S with original PCMS-1. XRD patterns of PCMS-1-O and PCMS-1-S show typical diffraction peaks of ZnS with no detectable ZnO (Fig. S11).


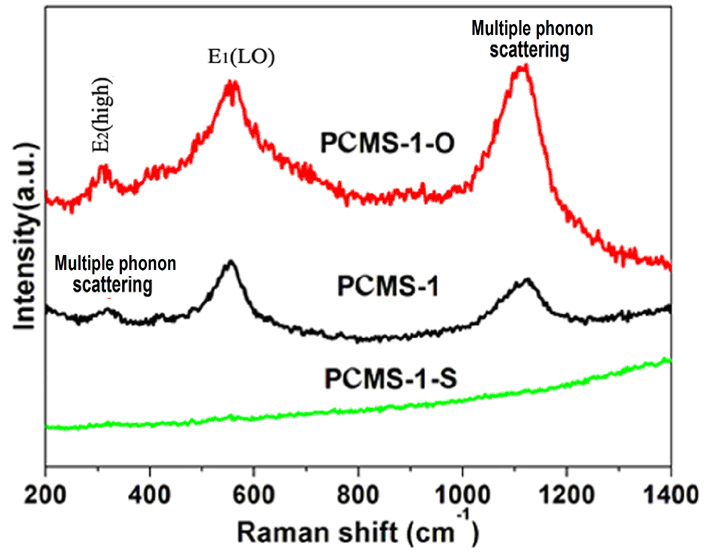


**Fig. S12** Raman spectra of PCMS-1, PCMS-1-S (prepared by treatment of PCMS under H_2_S atomsphere), PCMS-1-O (prepared by the treatment of PCMS under air). No vibration of Zn-O is found in Raman spectrum of PCMS-1-S, implying a lower ZnO content than that in PCMS-1.The PCMS-1-O gives the enhanced peaks of Zn-O vibration, indicating the increase of ZnO amount after the PCMS-1 is treated under air.


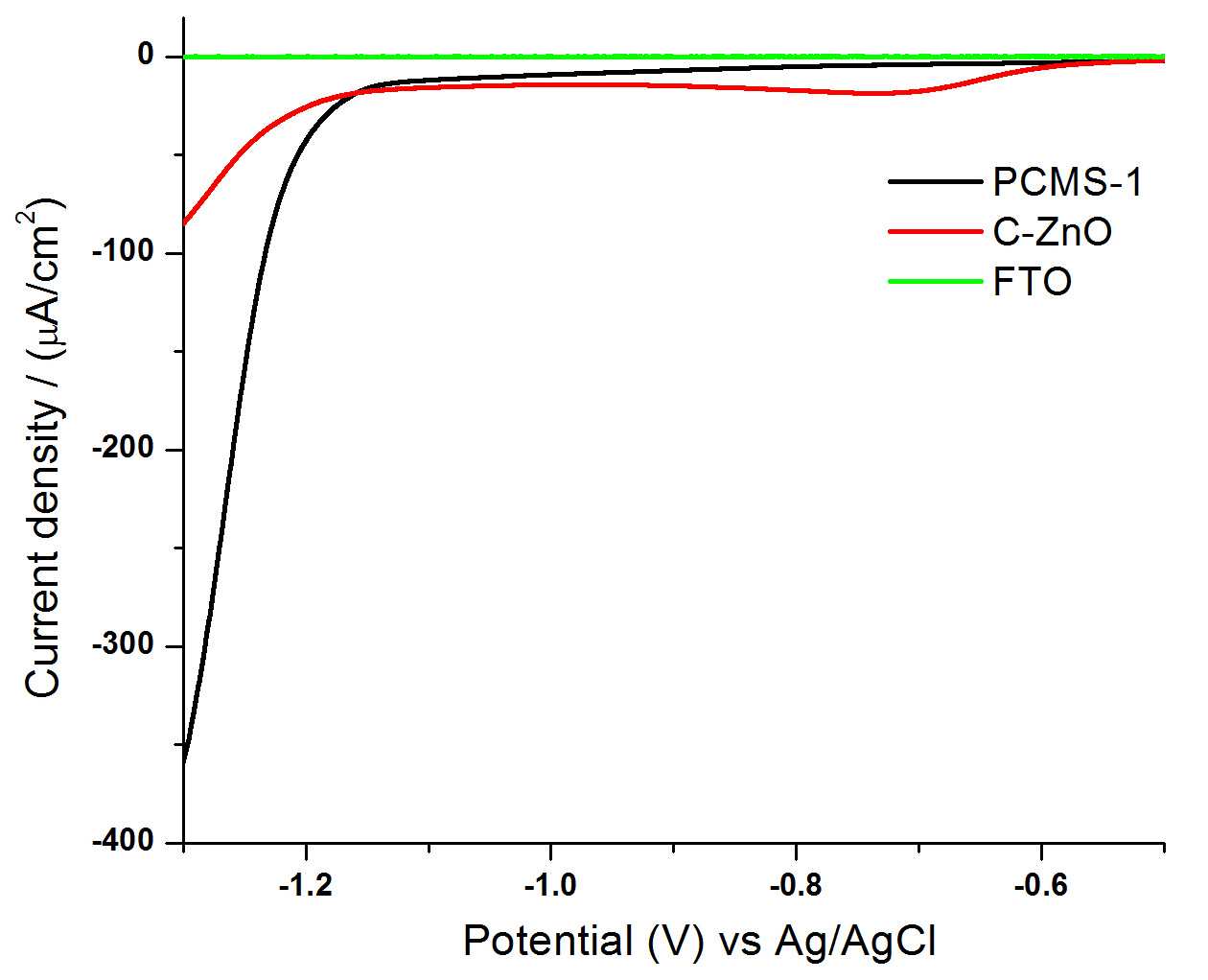


**Fig. S13** Current-voltage curves of C-ZnO and PCMS-1 on FTO and bare FTO in 0.5 M Na_2_SO_4_ solution.

.

Fig. S13 shows the current-voltage curves of C-ZnO (commerical ZnO) and PCMS-1 on FTO and bare FTO in 0.5 M Na_2_SO_4_ solution. For bare FTO, no obvious current attributed to the reduction of water to H_2_ can be seen. For the C-ZnO, the cathodic current attributed to the reduction of water to H_2_ can be obseved in the potential range of -1.17 to -1.3 V vs Ag/AgCl with obvious current. The results indicated that the ZnO can catalyze the evolution of H_2_. In addition, there is a great increase in the cathodic current for PCMS-1 electrode with more positive potential for water reduction reaction (about -1.13 V). When the potential is set at -1.3 V versus Ag/AgCl, the current observed on the PCMS-1 was about 4 times higher than that of C-ZnO. The results above imply that the ZnO can catalyze the evolution of H_2_ and the formation of ZnO-dotted structure is more favourable for the evolution of H_2_.


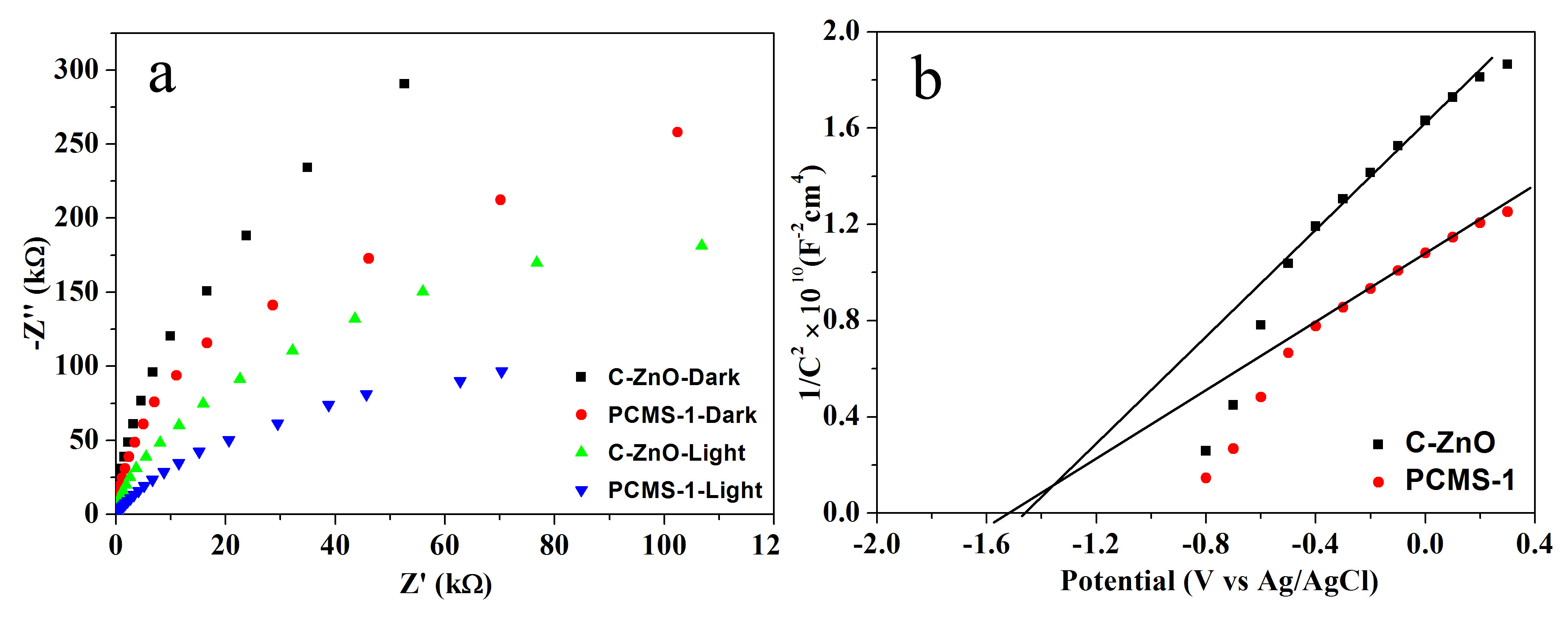


**Fig. S14** a) EIS and b) Mott-Schottky curve of C-ZnO and PCMS-1

In order to give further insight of charge transfer and separation, EIS and Mott-Schottky analysis were performed. The Nyquist plots of EIS in Fig.S14a displayed a semicircle at high frequency represents the charge-transfer process, and the diameter of the semicircle reflects the charge-transfer resistance. It is clearly observed that the diameters of the semicircle for PCMS-1 both in the dark and light are much smaller than those of commercial ZnO, ascribing into that S-Zn-O in PCMS-1 can act as electron channel which can promote the electron mobility by reducing the recombination of electron-hole pairs **(Ref.1)**. In addition, Mott-Schottky analysis in Fig. S14b clearly indicates the increasing of carrier density in PCMS-1. The carrier density (Nd) can be calculated from the slope of the Mott-Schottky plot using the following equation **(Ref.2)**.

$$N_{d}=(2/e_{0}\varepsilon\varepsilon_{0})[d(1/C^{2})/dV]^{-1}$$

where e_0_ is the electron charge, ε is the dielectric constant, ε_0_ is the permittivity of vacuum, Nd is the carrier density and V is the applied bias at the electrode. Importantly, PCMS-1 show smaller slopes of the Mott-Schottky plot compared to commercial ZnO, suggesting a higher carrier density.

Based on the above results of CV, EIS and Mott-Schottky, PCMS-1 have the fast electron mobility, high carrier density and positive potential for the evolution of H_2_, which should be relative with excellent PHE activity of PCMS sample.

**[1]** A. I. Kontos, V. Likodimos, T. Stergiopoulos, D. S. Tsoukleris and P. Falaras, *Chem. Mater.* **2009**, *21*, 662-672.

**[2]** F. Su, T. Wang, R. Lv, J. Zhang, P. Zhang, J. Lu, J. Gong, *Nanoscale* **2013**, *5*, 9001-9009.
